# Exploratory Study of Selective Brain Hypothermia Using Transnasal Evaporative Cooling Under Controlled Normothermia with an Endovascular Device

**DOI:** 10.3390/jcdd13030120

**Published:** 2026-03-06

**Authors:** Mitsuaki Nishikimi, Kazuya Kikutani, Mayumi Higashi, Shinichiro Ohshimo, Tatsuhiko Anzai, Nobuaki Shime

**Affiliations:** 1 Department of Emergency and Critical Care Medicine, Graduate School of Biomedical and Health Sciences, Hiroshima University, Hiroshima 734-8551, Japan; kikutani@hiroshima-u.ac.jp (K.K.); higashim@hiroshima-u.ac.jp (M.H.); ohshimos@hiroshima-u.ac.jp (S.O.); shime@koto.kpu-m.ac.jp (N.S.); 2 Department of Emergency and Critical Care Medicine, Aichi Medical University, Nagakute 480-1195, Japan; 3 Department of Biostatistics, Institute of Science Tokyo, Tokyo 113-8510, Japan; tanzai.dsc@tmd.ac.jp

**Keywords:** targeted temperature management, brain cooling, cardiac arrest, animal study, swine

## Abstract

Introduction: Selective brain hypothermia has been investigated to improve neurological outcomes in patients with cardiac arrest; however, an optimal clinical method has not yet been established. This study aimed to evaluate the feasibility of a technique combining transnasal evaporative cooling with simultaneous endovascular temperature management to achieve selective brain hypothermia while preventing systemic hypothermia. Methods: Three adult male Göttingen swine were anesthetized and mechanically ventilated. Transnasal cooling was initiated at maximum output while endovascular warming preserved systemic temperature. Brain parenchymal and rectal temperatures, mean arterial pressure (MAP), heart rate (HR), and cardiac output (CO) were continuously monitored for 60 min. Temperature differences between brain and rectum at 60 min were analyzed. Results: A brain–rectal gradient ≥1.0 °C was achieved in all swine at 25, 40, and 30 min, respectively, and maintained at 1.0–1.5 °C thereafter. Brain temperature (34.5 ± 0.34 °C) was significantly lower than rectal temperature (35.8 ± 0.35 °C) at 60 min after initiation of the selective cooling procedure (*p* = 0.0048). MAP, HR, and CO showed no deviations from baseline. Conclusions: The combination of transnasal cooling and endovascular warming reliably induced selective brain hypothermia of 1–1.5 °C without adverse effects on hemodynamic parameters in swine.

## 1. Introduction

Fever after cardiac arrest can exacerbate brain damage in patients with post-cardiac arrest syndrome (PCAS) [1]. To mitigate this risk, therapeutic hypothermia, which is also called targeted temperature management (TTM) at a lower setting temperature, was widely adopted as standard care for patients with PCAS after two landmark trials demonstrated improved neurological outcomes [2,3]. However, the subsequent large randomized trials (TTM-1 and TTM-2) failed to demonstrate a significant difference in favorable neurological outcomes between therapeutic hypothermia and controlled normothermia [4,5]. Based on these findings, current guidelines emphasize active prevention of fever [6]. Nevertheless, current guidelines do not completely reject hypothermia. They state that evidence remains insufficient to recommend for or against targeting 32–36 °C in specific patient subgroups and also discuss the potential role of early cooling strategies [6]. Thus, contemporary recommendations do not preclude investigation of alternative approaches aimed at reducing cerebral temperature.

Numerous experimental studies in animal models have consistently shown beneficial effects of therapeutic hypothermia on neurological outcomes after cardiac arrest [7,8]. However, these experimental benefits have not translated into improved outcomes in large clinical trials. Several factors may account for this discrepancy, including differences in the timing of cooling, the quality and timing of resuscitation, and baseline prognostic factors such as rates of bystander CPR. Among these possibilities, one important explanation is the potential adverse systemic effects of whole-body hypothermia. While lowering brain temperature can attenuate neuronal injury, systemic hypothermia possibly decreases cardiac output, increases systemic vascular resistance, and ultimately reduces cerebral blood flow, thereby attenuating or even counteracting its neuroprotective benefit [9,10].

Selective brain hypothermia has been proposed as a strategy to overcome these limitations by cooling the brain while maintaining systemic normothermia [11]. Although several techniques have been investigated, no clinically established and practical method for selective brain cooling currently exists [12].

The aim of this study was to evaluate a novel selective brain cooling strategy in healthy swine that combines a transnasal cooling device with an endovascular temperature management device. The transnasal cooling device achieves rapid evaporative cooling by spraying an inert coolant into the nasal cavity [13]. By utilizing the highly vascular nasal cavity (conchae and turbinates), which lies in close proximity to the cerebral circulation, nasopharyngeal cooling can preferentially lower brain temperature before systemic temperature falls [14]. Although the effects of transnasal cooling to hemodynamic parameters in patients with PCAS have not been well characterized [15], this technique is known to ultimately decrease systemic temperature through the circulation of cooled blood [16] and therefore could plausibly influence hemodynamic status. In principle, this tendency to lower systemic temperature could be counteracted by intravascular temperature management using the endovascular device. Then, we hypothesized that brain temperature could be selectively reduced with transnasal cooling while limiting excessive systemic cooling through endovascular temperature management. This novel approach requires no specialized techniques, and all devices are already available in clinical practice.

## 2. Materials and Methods

### 2.1. Chemicals

All chemicals were obtained from FUJIFILM Wako Pure Chemicals (Osaka, Japan).

### 2.2. Animal Preparation

The Committee of Animal Experimentation of Hiroshima University approved the protocol (A23-140, 4 January 2024). Experiments were conducted at the Natural Science Center for Basic Research and Development, Hiroshima University. Three adult male Göttingen swine (aged ≥ 10 months, approximately 30 kg; Hiroshima Experimental Animal Research Center) were used. Animals were fasted overnight. Sedation was induced with intramuscular ketamine (4 mg/kg) and midazolam (0.5 mg/kg) before transfer to the operating room. After venous access was secured via the auricular vein, continuous intravenous propofol (3 mg/kg) was initiated. Tracheostomy was performed, and a cuffed 7 mm tracheal tube was inserted and inflated to prevent gas leakage and aspiration.

During the entire experiment, anesthesia and analgesia were maintained with midazolam (0.5–0.8 mg/kg/h IV), ketamine (2.5–7.5 mg/kg/h IV), and fentanyl (5–15 mcg/kg/h IV). Animals were mechanically ventilated using a pressure-controlled ventilator, with peak inspiratory pressure adjusted to achieve tidal volumes of 10–15 mL/kg and positive end-expiratory pressure of 5 cm H_2_O. The fraction of inspired oxygen was adjusted to maintain SpO_2_ at 94–98%.

A femoral arterial catheter was inserted for the measurement of hemodynamic parameters, namely mean arterial pressure (MAP), heart rate (HR), and cardiac output (CO). CO was calculated from arterial waveform analysis (PulsioFlex PC4000^®^, Getinge, Göteborg, Sweden). Because the monitoring system was designed for humans and required height and body surface area inputs not applicable to swine, CO was reported as a ratio to each animal’s baseline value.

An endovascular temperature control catheter (Quattro^®^, Zoll, Chelmsford, MA, USA) was inserted via the femoral vein for systemic warming and drug administration. Core body temperature was measured using a rectal probe.

Brain parenchymal temperature was recorded using an intracranial catheter thermometer (Pressio^®^ Catheters, Sophysa, Eden Prairie, MN, USA). The surgical procedure for intracranial temperature monitoring followed previously described methods [17,18]. After a right paramedian incision, a small burr hole was created over the frontal cortex, located 5–15 mm lateral to the midline and 5–10 mm anterior to the coronal suture. The intracranial catheter thermometer was advanced into the brain parenchyma to a depth of approximately 1–1.5 cm.

**Figure 1:**
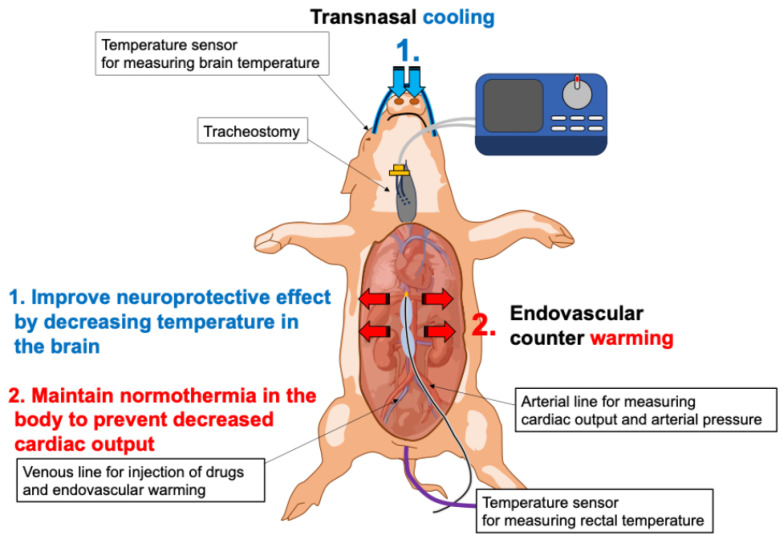
Schema of the novel selective brain hypothermia procedure.

Rectal temperature was measured as an index of core body temperature, consistent with actual clinical practice during TTM [19]. A transnasal cooling device (RhinoChill^®^, BrainCool, San Diego, CA, USA) was placed in the nasal cavity. This device delivers compressed air/oxygen to spray a liquid coolant into the patient’s nostrils which vaporizes the upper surface of the nasal cavity, absorbing heat from the tissue. The schema of animal experimental setting is illustrated in Figure 1.

### 2.3. Selective Brain Hypothermia Under Controlled Normotharmia

Before selective cooling, core body temperature was stabilized at 36.0 ± 0.5 °C for ≥1 h using an electric warming blanket and temperature control system through endovascular catheter (Thermoguard^®^, Zoll, USA). Active cooling was then initiated through the transnasal device at maximum output, while concurrent rewarming through the endovascular catheter targeted 38.0 °C to maintain normothermia. Brain and rectal temperatures, MAP, HR, and CO were monitored continuously for 1 h. At the end of the experiment, anesthesia was deepened with additional intravenous ketamine (5 mg/kg) and propofol (5 mg/kg, titrated to effect). Adequate depth of anesthesia was confirmed, and the animals were then euthanized by exsanguination.

## 3. Primary Outcome

The primary endpoint was a sustained brain–rectal temperature gradient ≥1.0 °C.

### 3.1. Sample Calculation

Sample size was calculated by using two paired means. For the primary outcome measure, we assumed a mean difference between brain and rectal temperature at 60 min after the start of selective cooling of 1.0 °C, which we considered as a minimum clinically meaningful effect size, with a standard deviation of 0.3 based on previous reference [20]. It was estimated that a sample size of three per group would be needed, at least, to obtain at least 80% statistical power at a two-sided significance level of 5% by the paired *t*-test.

### 3.2. Statistical Analysis

Continuous variables were expressed as mean ± standard deviation. Brain and rectal temperatures at 60 min after initiation of selective cooling were compared using the paired *t*-test. Also, a linear mixed-effects model was used to compare brain and rectal temperatures over time from 0 to 60 min (5 min intervals) after initiation of selective cooling, including a time-by-region interaction term. *p* values < 0.05 were considered statistically significant. Analyses were performed using JMP software (version 17.0, SAS Institute Inc., Cary, NC, USA).

## 4. Results

Three swine were used in this study. Baseline characteristics are presented in Table 1. The duration of surgery, which was defined as the time from induction of anesthesia to the initiation of selective brain hypothermia, was 104 min for the first swine, 112 min for the second, and 93 min for the third.

**Figure 2:**
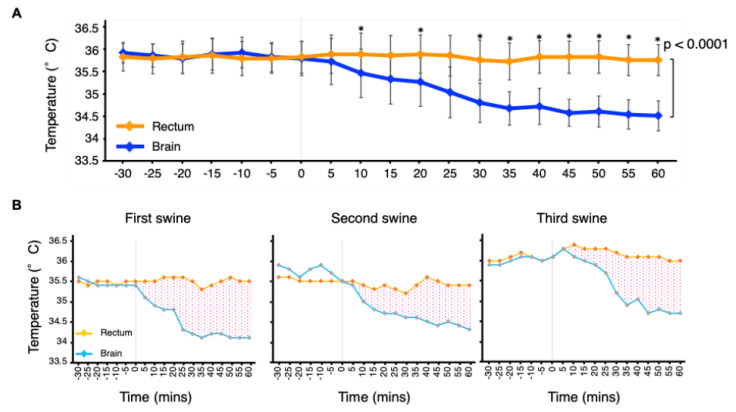
Rectal and brain temperature changes during the observational period. (**A**) illustrates overall rectal (orange) and brain (blue) temperature changes. Time 0 indicates initiation of selective brain cooling. Time course of mean brain and rectal temperatures. Data are shown as mean ± SD for three swine with 5 min intervals. A linear mixed-effects model with a time-by-region interaction term demonstrated a significant difference in temporal trends between brain and rectal temperatures (*p* < 0.0001). Asterisks indicate time points at which paired *t*-tests showed a significant difference (*p* < 0.05) between brain and rectal temperatures. The exact *p* values are shown in Table 2. (**B**) depicts rectal and brain temperature changes in three individual swine. Red dots express the difference between rectal and brain temperatures after starting selective brain cooling.

Changes in rectal and brain temperatures from 30 min before to 60 min after initiation of the selective cooling procedure are shown in Figure 2A. Brain temperature declined rapidly after cooling began, while rectal temperature remained close to its baseline. Consequently, the brain–rectal gradient widened immediately, plateaued at approximately 45 min, and was maintained at 1.0–1.5 °C during the subsequent 15 min (Figure 2A). The time required to achieve a 1.0 °C gradient was 25, 40, and 30 min for the first, second, and third swine, respectively (Figure 2B). Paired *t*-tests showed that brain temperature (34.5 ± 0.34 °C) was significantly lower than rectal temperature (35.8 ± 0.35 °C) at 60 min after initiation of the selective cooling procedure (*p* = 0.0048). A linear mixed-effects model including a time-by-region interaction term (brain vs. rectum) demonstrated a significant interaction, indicating different temporal trends in rectal and brain temperatures over the 0–60 min observation period (*p* < 0.0001). Pointwise paired *t*-tests showed that rectal temperatures were significantly higher than brain temperatures at all time points from 30 min after initiation of selective cooling (Table 2). These results support that a brain–rectal temperature gradient was sustained during this period.

**Figure 3:**
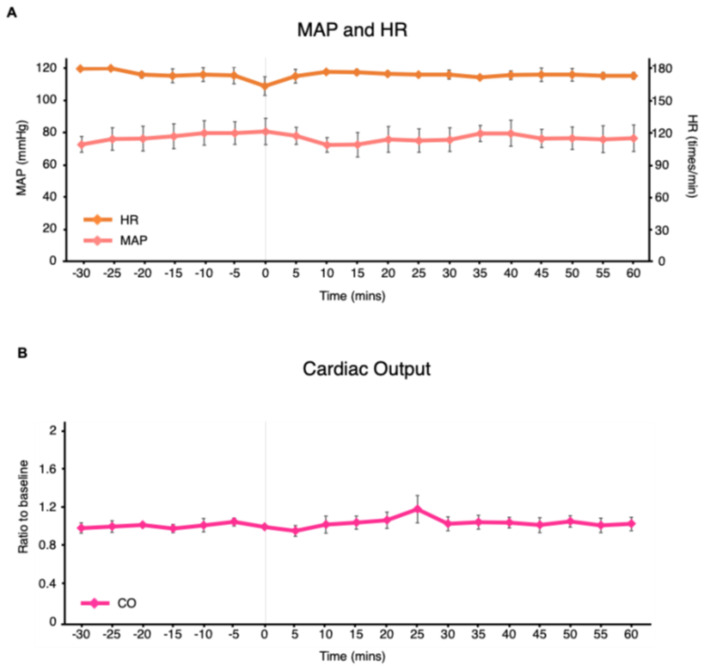
Mean arterial pressure, heart rate, and cardiac output during the observational period. (**A**) Time course of mean arterial pressure and heart rate from 30 min before to 60 min after initiation of selective brain cooling (time 0). Data are shown as mean ± SD for three swine, measured at 5 min intervals. (**B**) Time course of cardiac output from 30 min before to 60 min after initiation of selective brain cooling (time 0). Data are shown as mean ± SD for three swine, measured at 5 min intervals; MAP, mean arterial pressure; HR, heart rate; CO, cardiac output.

The effects of selective brain hypothermia on hemodynamic parameters were minimal. At baseline, MAP and HR were stable (60–80 mmHg and 170–180 bpm, respectively). Following initiation of selective brain hypothermia, both parameters remained unchanged throughout the observation period (Figure 3A). CO was also stable, consistently ranging between 0.95 and 1.05 of baseline throughout the observation period (Figure 3B).

## 5. Discussion

This study developed a novel method for selective brain hypothermia using transnasal cooling combined with endovascular warming. The approach consistently achieved a brain–rectal gradient of 1.0–1.5 °C without hemodynamic compromise.

Brain temperature decreased by 1.0–1.5 °C within 45 min, a physiologically meaningful reduction. Hypothermia lowers cerebral metabolic rate and brain oxygen consumption by approximately 6–7% for each 1.0 °C decrease in temperature [21]. Previous animal studies have shown that a 1.0–2.0 °C reduction in brain temperature may prevent ischemic injury [22,23]. In contrast, a 2.0 °C reduction in systemic temperature markedly impairs left ventricular contractile function [24]. Our model maintained body temperature ≥ 35.5 °C while lowering brain temperature, demonstrating effective selective cooling without systemic adverse effects.

Although the target temperature for endovascular warming was set at 38 °C, systemic core temperature remained at approximately 36 °C, indicating that intranasal cooling exerted a measurable systemic cooling effect. In our configuration, blood actively warmed in the inferior vena cava by the endovascular catheter returned to the right atrium and mixed with cooled venous blood primarily entering through the superior vena cava. The observed core temperature therefore likely represents a physiological equilibrium between simultaneous systemic warming and cooling. Despite this systemic effect, a brain–core temperature gradient was consistently maintained. This observation can be explained by the cooling characteristics of intranasal evaporative cooling. Local heat exchange at the nasal cavity and skull base allows preferential cooling of arterial inflow to the brain via the cavernous sinus and carotid circulation, resulting in a greater and more rapid temperature reduction in cerebral tissue than in the rest of the body.

A key strength of this method is its reliance on commercially available, clinically approved devices. Both the transnasal cooling system and the endovascular temperature-management catheter have established safety profiles in patient populations [25,26]. This makes the approach feasible for translation into clinical research. Nevertheless, further prospective studies are necessary to establish standardized protocols or explore more appropriate methods. For example, in our protocol we initiated brain cooling with the transnasal device and endovascular warming simultaneously. It is possible that brain temperature could be reduced more rapidly if endovascular warming were started at a later time point. Also, given that the transnasal cooling device can typically be set up more quickly than the endovascular system, one potential approach would be to start with transnasal cooling alone and introduce endovascular warming only if hemodynamic insufficiency occurs.

This study had some limitations. We sought to generate meaningful data with a minimal number of animals, consistent with the 3Rs of animal research—Replacement, Reduction, and Refinement. Consequently, statistical power was limited, as is common in large-animal investigations [27], where costs of acquisition, husbandry, and monitoring, restrict sample size. Second, a control group without endovascular temperature management was not included. Because this experiment was designed as a pilot feasibility study to determine whether a brain–core temperature gradient could be maintained, each animal served as its own control. Future comparative studies, including an intranasal-cooling-only group, are required to quantify the contribution of endovascular temperature management. Third, we did not perform any post-intervention follow-up or histological assessment. Although both the transnasal cooling device and the endovascular catheter are already used in clinical practice, future studies are needed to comprehensively assess the safety and functional consequences of this combined selective brain hypothermia approach [15,28,29]. Fourth, direct measurement of brain temperature in this study required placement of an intraparenchymal thermometer, which is not feasible for routine clinical use. Therefore, real-time monitoring of cerebral temperature during combined intranasal cooling and endovascular warming remains a practical challenge. However, previous studies have suggested that tympanic membrane temperature may serve as a surrogate marker of cerebral temperature [30]. In addition, emerging non-invasive monitoring technologies may allow estimation of brain temperature without invasive probes [31]. Finally, the approach was not tested in disease models such as ischemic stroke or cardiac arrest. The effectiveness of selective cooling in pathological states, particularly where post-injury fever is prevalent, requires evaluation in validated ischemia/reperfusion and cardiac arrest models.

## 6. Conclusions

The combination of transnasal cooling and endovascular warming reliably induced selective brain hypothermia of 1–1.5 °C without adverse effects on hemodynamic parameters in swine. This simple, clinically applicable approach warrants further investigation in pathological models.

## Figures and Tables

**Table 1:** Baseline characteristics of the three swine used in this study.

Variable	First Swine	Second Swine	Third Swine
Body weight, kg	24	38	33
Surgical procedure time, min	104	112	93
Before experiment			
MAP, mmHg	63	75	80
HR, bpm	180	180	176
RR, times/min	12	14	15
SpO_2_, %	99	97	94
Rectal temperature, °C	35.5	35.6	36.5
Brain temperature, °C	35.6	35.9	36.4

All values represent single baseline measurements obtained at one time point in each swine. MAP, mean arterial blood pressure; HR, heart rate; RR, respiratory rate.

**Table 2:** Comparisons of rectal and brain temperatures over the 60 min observation period.

Mixed Effect Model	*p*_interaction_ < 0.0001
Pointwise paired *t*-test	*p* values
0 min	0.4226
5 min	0.2999
10 min	0.0390
15 min	0.0669
20 min	0.0342
25 min	0.0702
30 min	0.0413
35 min	0.0153
40 min	0.0016
45 min	0.0048
50 min	0.0198
55 min	0.0094
60 min	0.0048

A linear mixed-effects model including an interaction term between time and measurement region (brain vs. rectum) was performed. The significant interaction indicates that the temporal trend in rectal temperature differed from that in brain temperature over the 0–60 min period. Pointwise paired *t*-tests at each time point showed that rectal temperatures were significantly higher than brain temperatures at all time points from 30 min after initiation of selective cooling.

## Data Availability

Data is available upon reasonable requests and in strict accordance with funding guidelines.
